# Lobeglitazone and Its Therapeutic Benefits: A Review

**DOI:** 10.7759/cureus.50085

**Published:** 2023-12-06

**Authors:** Balamurugan M, Sarumathy S, Robinson R

**Affiliations:** 1 Department of Pharmacy Practice, SRM College of Pharmacy, Faculty of Medicine and Health Sciences, SRM Institute of Science and Technology, Kattankulathur, IND

**Keywords:** pioglitazone, insulin resistance, lobeglitazone, thiazolidinediones, type 2 diabetes mellitus

## Abstract

Lobeglitazone is a newer oral hypoglycemic agent that has been tested in type 2 diabetes mellitus (T2DM). We aim to conduct a narrative review to find out the therapeutic benefits of lobeglitazone in patients with T2DM. We scientifically searched the electronic database of PubMed from inception until September 12, 2023, using Medical Subject Heading (MeSH) keywords. Additionally, we searched for pre-clinical trials related to lobeglitazone. We retrieved all available results of phase 1 to phase 3 studies of lobeglitazone in T2DM. Subsequently, we reviewed the results narratively. Three double-blind, randomized, placebo-controlled studies and a phase 3 trial of lobeglitazone showed that 0.5 mg daily dose exhibits effective therapeutic activity in glycemic, lipid, and hepatic control, and is also used as a secondary treatment in non-alcoholic fatty liver disease. Lobeglitazone exhibits as much antidiabetic activity as other thiazolidinediones such as pioglitazone and rosiglitazone. Side effects of lobeglitazone included peripheral edema, weight gain, and bone mineral density, which did not require hospitalization for these effects. This article highlights the pharmacological, pre-clinical, clinical, and safety pharmacology of novel thiazolidinedione lobeglitazone.

## Introduction and background

Insulin resistance and aberrant cells are two features of the metabolic disease type 2 diabetes mellitus (T2DM) [1]. In patients with T2DM, thiazolidinediones (TZDs) are the first oral hypoglycemic medications that increase skeletal muscle insulin sensitivity and decrease hepatic glucose synthesis by activating peroxisome proliferator-activated receptor gamma (PPAR-γ) agonists [2]. The nuclear receptor superfamily includes the peroxisome proliferator-activated receptor (PPAR). It performs as a ligand-activated transcription factor and is crucial for controlling inflammation, insulin resistance, and adipocyte differentiation [1]. For the treatment of T2DM, rosiglitazone and pioglitazone are the most often utilized TZDs. However, because of its negative side effects, including edema, cardiac issues, and the risk of bladder cancer, their use has declined. The need for a powerful and secure TZD was met by the recently discovered drug lobeglitazone [1]. Some Asian countries such as Korea and India have approved the use of lobeglitazone for the treatment of T2DM. It was invented and approved in Korea [3]. Pioglitazone has a positive impact on lipid profiles in T2DM patients. Both in vitro and in vivo studies demonstrate that lobeglitazone is more effective than the other TZD drugs (such as pioglitazone and rosiglitazone) [2]. According to previous research, lobeglitazone medication improved lipid profiles by an 8% rise from baseline high-density lipoprotein cholesterol (HDL-C) levels and a 13% reduction from baseline triglycerides levels [2]. Both as a monotherapy and in combination with metformin, lobeglitazone was well tolerated. Because of its greater affinity for PPAR, lobeglitazone exhibits comparable glycemic efficiency to pioglitazone at a lower effective dose of 0.5 mg [4]. It is anticipated that lobeglitazone can be administered in individuals with renal insufficiency without dose reduction since it is mostly metabolized by the liver, with minor renal excretion, and because it may have a lower risk of bladder cancer than other TZDs [1]. Due to its favorable safety profile and proven ability to decrease blood sugar levels in both clinical and experimental studies, Korea has approved lobeglitazone as an oral diabetes medication since July 2013 [5]. According to research conducted on animals, lobeglitazone administration greatly decreased insulin resistance and enhanced the expression of GLUT4 and PPARs in mice given a high-fat diet (HFD) [6]. The prevalence of diabetes is significantly rising globally, primarily as a result of rising obesity and insulin resistance rates as well as an aging population that has an impact on mortality and quality of life [7].

Lobeglitazone was initially invented by Chong Kun Dang Pharmaceutical in Korea to treat diabetes. On July 4, 2013, the Ministry of Food and Drug Safety (Korea) approved lobeglitazone, i.e., the pharmaceutical product that Chong Kun Dang Pharmaceutical markets under the brand name Duvie® [8]. Duvie is offered as an oral tablet that contains 0.5 mg of free lobeglitazone. The suggested dosage is 0.5 mg administered once daily [8]. Indians have a high rate of insulin resistance, which is why Glenmark Pharmaceuticals Limited was the first company to launch lobeglitazone in India for the treatment of type 2 diabetes. The drug is marketed under the trade name LOBG and contains lobeglitazone (0.5 mg), which is to be taken by patients orally once daily. It is prescribed to improve glycemic control in patients with insulin resistance. As per the FDA reports, Glenmark previously received approval from India's drug regulator, the Drug Control Directorate General of India (DCGI), to manufacture and market lobeglitazone, based on a randomized, double-blind trial in stage 3 patients/individuals aged 18 years and over who had type 2 diabetes. The results of this trial showed faster and improved glycemic control with lobeglitazone. Few preclinical studies have reported beneficial effects of lobeglitazone in improving blood glucose levels, lipid profile, atherosclerosis, renal fibrosis, and non-alcoholic fatty liver disease (NAFLD) [1].

## Review

Chemistry of lobeglitazone

The molecular formula of lobeglitazone is C24H24N4O5S. Its synonyms include Duvie (Korea) and LOBG (India). Its molecular weight is 480.5 g/mol. Its IUPAC (International Union of Pure and Applied Chemistry) name is 5-[4-(2-{[6-(4-Methoxy-phenoxy)-pyrimidine-4-yl]-methyl amino}-ethoxy)-benzyl]-thiazolidine-2,4-dione hydro sulfuric acid.

**Figure 1:**
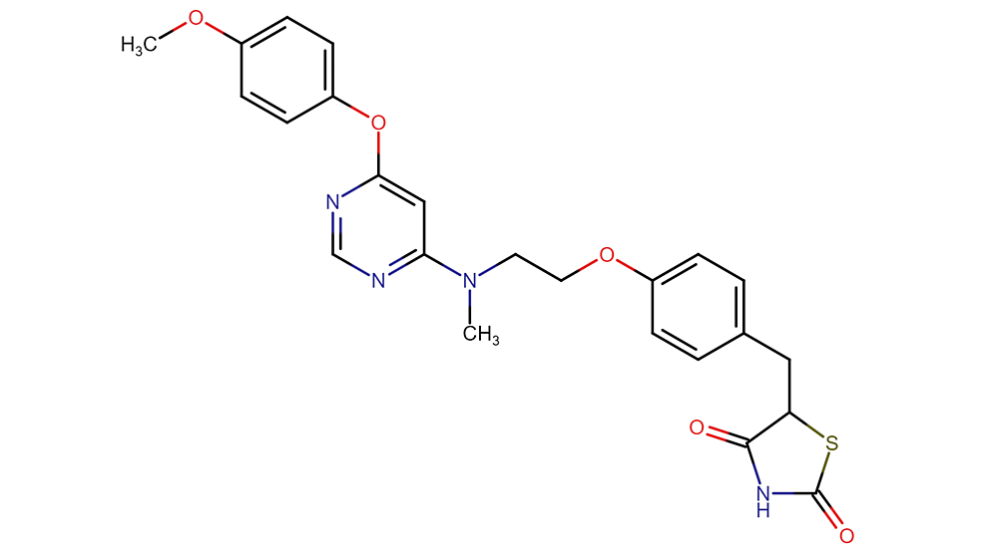
The structure of lobeglitazone drawn using Chemspace (https://chem-space.com)

Lobeglitazone was developed by introducing a p-methoxy phenoxy group at the fourth position of the pyrimidine group in the rosiglitazone structure. This is the reason lobeglitazone has a high binding affinity to PPAR-γ. Lobeglitazone has a 12-fold higher affinity than rosiglitazone and pioglitazone (Figure 1).

Pharmacokinetics

The pharmacokinetics of lobeglitazone have been observed in healthy volunteers by Kim et al. [1]. The study was performed in healthy male subjects. Following oral administration of a single dose of lobeglitazone, peak plasma concentrations are reached in one to three hours and then decrease exponentially with a half-life of 7.8-9.8 hours. In men, peak plasma concentrations (C max) and area under the curve (AUC) from time 0 to infinity increased dose proportionally over the dose range 1 to 4 mg. After multiple doses of lobeglitazone, steady-state concentrations were reached on day five and no clinically significant accumulation was observed [9]. Another study was performed on healthy female subjects after a single dose. The systemic exposure to lobeglitazone after 2 mg was similar to that in men, but after a 4 mg dose, the exposure was 2.36 times higher in women than in men [2]. Lobeglitazone had no meaningful effect on the pharmacokinetics or pharmacodynamics of warfarin [10]. Lobeglitazone and metformin alone had steady-state maximum plasma concentrations; C(max, ss) and mean ± standard deviation of 29.38 ± 5.25 ng/mL and 1661.84 ± 471.88 ng/mL, respectively; when co-administered, the C(max, ss) were 27.15 ± 5.75 ng/mL and 1779.92 ± 405.20 ng/mL, respectively. When both medications were taken together, lobeglitazone did not substantially alter the pharmacokinetics of metformin or the other way around. Metformin and lobeglitazone can be taken together without changing the dosage of either medication [11]. For lobeglitazone administered concurrently with amlodipine versus lobeglitazone administered alone, the geometric mean ratio (with 90% CIs) of Cmax,ss and AUCτ,ss was 1.01 (0.93-1.09) and 1.06 (0.92-1.23), respectively. The Cmax,ss and AUCτ,ss geometric mean ratios (with 90% CIs) for amlodipine given concurrently with lobeglitazone compared to amlodipine given alone were 0.98 (0.94-1.02) and 1.00 (0.96-1.05), respectively. In healthy male Korean patients, the coadministration of lobeglitazone and amlodipine had no effect on the pharmacokinetics of either drug [12].

Pharmacodynamics

The TZD portion of lobeglitazone occupies the canonical ligand-binding pocket near the activated functional helix-2 (AF-2) (i.e., helix H12) in the ligand-binding domain as well as the TZD fragment of rosiglitazone. However, the elongated p-methoxy phenol moiety of lobeglitazone interacts with the hydrophobic pocket, enhancing its binding affinity and possibly affecting the cyclin-dependent kinase 5 (Cdk5)-mediated phosphorylation of PPAR-γ in ser245 (in PPAR-γ-1 numbering; ser273 in PPAR-γ-2 numbering). Lobeglitazone increased PPAR-γ phosphorylation at ser245 in a dose-dependent manner and exhibited a better inhibitory effect on ser245 phosphorylation than rosiglitazone [13]. Binding analysis using constructs of PPAR-γ bound to TZD showed that lobeglitazone exhibits a 12-fold higher affinity for PPAR-γ than rosiglitazone and pioglitazone. This structural difference correlates with increased affinity and low effective dose of lobeglitazone compared with other TZDs. Because of lobeglitazone's notable improvements in binding affinity and specificity, it appears that a low effective dose is possible, which lessens the negative effects brought on by off-target effects and chemical-based toxicity. The structural basis for PPAR-γ-TZD interactions is provided by this work, which also explains why lobeglitazone has a higher affinity than other TZD medications [14].

Results

Blood Lipid Effects

A study conducted by Sin Gon Kim et al. showed that treatment with lobeglitazone for 24 weeks improved blood levels of triglycerides, HDL-C, and low-density lipoprotein cholesterol (LDL-C) (small-dense LDL-C) [2]. The study conducted by Sun Hwa Kim et al. showed statistically significant improvements in small-dense triglyceride and LDL-C over a 28-week duration [15]. SM Jin et al. concluded that lobeglitazone, as an adjunct to metformin, showed a significant reduction in HDL-C levels and a decrease in free fatty acids and triglycerides [4].

Non-alcoholic Fatty Liver Disease

A study conducted by Naga Chalasani et al. concluded that pioglitazone achieved the secondary goal of resolving NAFLD, achieved in a higher number of patients receiving pioglitazone than in the placebo group [16]. Young Ho Lee et al. observed that lobeglitazone reduced liver fat content, as assessed by a controlled attenuation parameter (CAP) and used to noninvasively quantify liver fat content. Lobeglitazone treatment significantly (P < 0.01) reduces the aspartate aminotransferase (AST), alanine transaminase (ALT), and gamma-glutamyl transpeptidase (GGTP) [17].

Ischemic Stroke

Yoo et al. conducted a study on diabetic patients who had an ischemic stroke, as these patients were at increased risk for stroke and cardiovascular complications. Lobeglitazone reduces the risk of cardiovascular complications in diabetic ischemic stroke patients similarly to other pioglitazone TZDs without increasing the risk of heart failure [5].

Albuminuria

A randomized controlled trial (RCT) conducted by Kim et al. on albuminuria in patients with type 2 diabetes concluded that lobeglitazone improved albuminuria in patients with type 2 diabetes as measured by the urinary albumin-creatinine ratio (UACR), and demonstrated it as beneficial. In the lobeglitazone treatment group, the new onset of microalbuminuria was reduced by 73%, the progression of albuminuria was reduced by 52%, and there was no significant difference with the pioglitazone treatment group [3].

Blood Sugar Control

In Sin Gon Kim et al.'s RCT performed 24 weeks after discontinuation of lobeglitazone in the efficacy analysis set (i.e., the intention-to-treat population), the lobeglitazone group was significantly more effective than the placebo group in achieving the target of glycosylated hemoglobin (HbA1c) <7% (44% vs. 12%, p < 0.0001). In the lobeglitazone group, fasting plasma glucose (FPG) (p < 0.0001), homeostasis model assessment of insulin resistance (HOMA-IR) (p = 0.002), and homeostasis model assessment of β-cell function (HOMA-β) (p = 0.0277), all showed improvements; in the placebo group, no changes were observed. Lobeglitazone 0.5 mg shows a favorable balance between safety and efficacy [2]. In the management of T2DM, negative regulation of insulin by the enzyme protein tyrosine phosphatase 1B (PTP1B) is considered a potential therapeutic target. An in vitro study concluded that lobeglitazone has reversible non-competitive inhibition of PTP1B [18]. Compared with other TZDs, lobeglitazone showed beneficial effects on beta-cell survival and control of hyperglycemia [19].

Corneal Fibroblasts

Nuwormegbe et al.'s study describes the synthesis and differentiation of extracellular matrix (ECM) impaired by transforming growth factor beta 1 (TGF-β1) by lobeglitazone and the differentiation of myofibroblasts from corneal fibroblasts. This anti-vibration effect appears to be independent of PPAR signaling. Lobeglitazone inhibited corneal fibroblasts' TGF-β1-induced ECM production and myofibroblast differentiation. This antifibrotic impact seemed to result from the suppression of TGF-β1-induced Smad signaling rather than PPAR signaling. Lobeglitazone also inhibited the transcription of nicotinamide adenine dinucleotide phosphate oxidase (Nox) 4 and the production of reactive oxygen species (ROS) produced by TGF-β1. The results concluded that lobeglitazone has a beneficial therapeutic effect on corneal scarring [20].

Pre-clinical studies

Min Jeong Kwon et al. studied the effects of different TZDs, including pioglitazone, rosiglitazone, troglitazone, and lobeglitazone. Beta-cell apoptosis and proliferation, ER stress markers, and glucose-stimulated insulin secretion (GSIS) were evaluated. In addition, metabolic parameters and islet profiles were also examined using C57BL/6 db/db mice treated with pioglitazone and lobeglitazone for four weeks. The results showed that lobeglitazone and other TZDs significantly reduced the rise in blood glucose caused by markers of ER stress and increased GSIS. The greatest improvement in metabolic parameters was in C57BL/6 db/db mice treated with pioglitazone and lobeglitazone compared with control mice [19]. A study conducted by Bong-Hoi Choi et al. evaluated the hypoglycemic effects of lobeglitazone and factors associated with lobular-enhanced fatty liver disease in rats fed an HFD. Mice were fed HFD for 15 weeks. The duration of oral lobeglitazone administration was nine weeks. The blood glucose levels of HFD-fed mice were considerably lower after nine weeks of Lobe 5 mg/kg/d. In HFD-fed mice, the effects of Lobe on serum glucose, insulin, and HOMA-IR levels were investigated. Lobe 5 mg/kg/d was sufficient to effectively reverse hyperglycemia and hyperinsulinemia in mice fed an HFD [6]. A study performed by Jong-Hwa Lee et al. determined the kinetics of absorption, distribution, metabolism, and excretion of lobeglitazone in rats. A cytochrome P450 (CYP) inhibitory study showed that lobeglitazone is mostly metabolized by the isoenzyme CYP3A4. Lobeglitazone exposure increases systemic exposure, which may cause certain adverse effects in people using a CYP3A4 inhibitor concurrently with lobeglitazone. Lobeglitazone interacts primarily with CYP1A2, 2C9, and 2C19 in the presence of lobeglitazone, and the substrate's metabolism was reduced by up to 41.0 ± 6.56%, 50.1 ± 5.71%, and 43.2 ± 3.71% for CYP1A2, 2C9, and 2C19, respectively. This rat study found that orally administered lobeglitazone was readily absorbed (absolute bioavailability ~95%). During intravenous administration, it results in linear pharmacokinetics over the dose range of 0.5 to 2 mg/kg. Lobeglitazone is mainly distributed in the liver, heart, lungs, and adipose tissues. Excretion of lobeglitazone via urine, bile, feces, and intestines was negligible (i.e., <10% of dose) in rats [21]. Dabin Jeong et al. conducted a preclinical trial of the anti-inflammatory activity of lobeglitazone in lipopolysaccharide-induced bone marrow-derived macrophages (BMDMs). Lobeglitazone inhibits nitric oxide (NO), which causes inflammation, and also inhibits other major inflammatory gene expressions in lipopolysaccharide-induced BMDM [22].

Safety pharmacology

Lobeglitazone has tolerability as a monotherapy and as a combination with metformin; the ratio of adverse events was similar to that of the placebo in the RCT [2]. Peripheral edema is one of the side effects associated with the TZDs. During 24 weeks of lobeglitazone monotherapy, peripheral edema was 3.6% in the lobeglitazone group vs. 0% in the placebo group. The incidence of peripheral edema was acceptable, based on clinical studies using pioglitazone monotherapy [1]. The pharmacokinetic profile and safety of lobeglitazone were studied on T2DM patients with hepatic impairment compared with control groups. It was found that lobeglitazone can be safely prescribed to patients for T2DM with mild or moderate hepatic impairment without dose adjustment [23]. Generally, TZDs are related to various safety concerns such as weight gain. The lobeglitazone group gained more weight at 24 weeks than the placebo group (0.89 kg vs. −0.63 kg, mean difference 1.52 kg; p < 0.0001). After treatment, there were no appreciable variations in the waist or ankle circumferences between the two groups (p = 0.06 and 0.14, respectively), bladder cancer, and congestive heart failure. Bo-Yeon Kim et al. concluded that lobeglitazone exhibits a long-term safety profile and effective glycemic control in clinical therapy [24]. Lobeglitazone has well-tolerated anti-diabetic therapy in healthy females. At lobeglitazone 2 mg, there is no sex difference in exposure to the drug, while lobeglitazone 4 mg has greater exposure of the drug in females than males. Min Kyu Park et al.'s study concludes no dose adjustment is required relating to sex differences for lobeglitazone because the glycemic control therapy is based on the individualized response to glycemic control [25]. Diabetic patients are at high risk of fractures independent of types of diabetes [26] at low bone mineral density (BMD) when compared to non-diabetics [27]. In diabetic conditions, increasing cortical porosity and decreasing the cortical area and bone material strength results in increased bone fragility leading to bone loss [28]. Lim et al. evaluated the impact of a 52-week course of lobeglitazone on BMD, which is known to be affected by TZD. The study concluded that no significant difference was observed in femoral neck BMD and total hip BMD between the lobeglitazone and placebo groups (Table 1) [29].

**Table 1:** Comparative safety and efficacy of thiazolidinediones such as lobeglitazone, pioglitazone, and rosiglitazone. PPAR-γ: peroxisome proliferator-activated receptor gamma; FBG: fasting blood glucose; HbA1c: glycosylated hemoglobin; HOMA-IR: homeostatic model assessment of insulin resistance; TG: triglyceride; HDL: high-density lipoprotein; LDL: low-density lipoprotein; Apo B: apolipoprotein B; PPAR: peroxisome proliferator-activated receptor.

Parameter	Lobeglitazone	Pioglitazone	Rosiglitazone
Molecular formula	C24H24N4O5S	C19H20N2O3S	C18H19N3O3S
PPAR-γ activity	12 times higher binding than pioglitazone and rosiglitazone [13]	Half maximal effective concentration of lobeglitazone for PPAR e4γ is 0.1374 µM versus pioglitazone 0.5492 µM [1]	Rosiglitazone has a higher affinity for PPAR gamma than troglitazone [30]
Potency	Maximum dose of 0.5 mg	Maximum dose of 45 mg	Maximum dose of 12 mg
Trial population	Korean	Worldwide	Worldwide
FBG reduction drug vs. placebo	150.91 → 131.80 mg/dl, P < 0.0001 [2]	172.6 → 143.8 mg/dl, P < 0.0001 [31]	227 → 189 mg/dl, P < 0.0001 [32]
HbA1c reduction drug vs. placebo	Difference = 0.6% for 0.5 mg (baseline HbA1c = 7.95%) [2]	Difference = 0.5% for 7.5 mg, 1% for 15 mg, 1% for 30 mg, 1.6% for 45 mg (mean baseline HbA1c: 10.4%) [24]	Difference = 0.3% for 2 mg, 0.6% for 4 mg (baseline HbA1c: 8.9%) [32]
HOMA-IR	3.89 → 2.69, P < 0.001 [4]	3.79 → 2.54, P < 0.001 [4]	1.73 reduction from the baseline value of HOMA-IR [33]
Lipid profile changes	TG 1.58 → 1.44 mmol/L, HDL 1.28 → 1.40 mmol/L, LDL 2.29 → 2.41 mmol/L, Apo B 0.72 → 0.79 g/dL [4]	TG 1.77 → 1.65 mmol/L, HDL 1.27 → 1.42 mmol/L, LDL 2.46 → 2.58 mmol/L, Apo B 0.76 → 0.84 g/dL [4]	TG 2.85 → 1.74 mmol/L, HDL 1.09 → 1.20 mmol/L, LDL 3.13 → 3.56 mmol/L [32]
Weight gain	+0.92 kg [4]	+0.76 kg [4]	+1.6 kg [32]
Edema	3.9% of all patients [4]	1.6% of all patients [4]	21.7% of all patients [32]

Ketoacidosis

Diabetic ketoacidosis (DKA) is one of the most serious complications of diabetes. It is characterized by the triad of hyperglycemia (blood sugar > 250 mg/dl), metabolic acidosis (arterial pH < 7.3 and serum bicarbonate < 18 mEq/L), and ketosis. The body becomes too acidic due to prolonged hyperglycemia. Ketoacidosis is one of the possible side effects of hyperglycemia [34].

The daily insulin dose is often reduced at the time of initiating sodium-glucose cotransporter-2 (SGLT2) therapy in insulin-treated patients to avoid hypoglycemia. However, reduction of insulin dose can increase the risk of ketoacidosis [35].

A study conducted by Muhammad Abdul-Ghani et al. shows dapagliflozin caused a four-fold increase in fasting plasma ketone concentration, while the combination of pioglitazone plus dapagliflozin was not associated with a significant increase (0.13 vs. 0.15 mM) in plasma ketone concentration or in risk of hypoglycemia. These results demonstrate that the addition of pioglitazone to dapagliflozin prevents the increase in plasma ketone concentration associated with SGLT2 therapy [35].

TZDs, a class of oral antidiabetic medications, have garnered attention for their potential role in the management of ketoacidosis, featuring notable representatives such as pioglitazone, rosiglitazone, and lobeglitazone [36]. Recent research has delved into the distinct mechanisms through which these drugs may confer beneficial effects in ketoacidosis scenarios. Pioglitazone, for example, has been investigated for its capacity to enhance insulin sensitivity and alleviate insulin resistance, thereby contributing to improved glucose utilization [35]. This effect is mediated through the activation of PPAR-γ, a pivotal transcription factor in glucose and lipid metabolism. Literature suggests that pioglitazone may alleviate ketoacidosis by addressing underlying insulin resistance, offering a potential adjunctive therapeutic approach to managing DKA [35,36]. Similarly, rosiglitazone has been explored for its potential role in ketoacidosis management, leveraging PPAR-γ activation akin to other TZDs [37]. Studies propose that rosiglitazone may enhance insulin sensitivity, improving glucose utilization and potentially mitigating insulin resistance, a critical factor in DKA development. By modulating gene expression in glucose and lipid metabolism, rosiglitazone extends its effects beyond glycemic control [37,38]. However, further clinical investigations are needed to delineate the specific impact of rosiglitazone in the context of ketoacidosis [38]. Lobeglitazone exhibits potential therapeutic effects in ketoacidosis through its action on PPAR-γ receptors [36]. Studies suggest that lobeglitazone may modulate inflammation and oxidative stress, contributing to the amelioration of DKA. The activation of PPAR-γ by lobeglitazone is implicated in reducing lipolysis and hepatic glucose production, leading to improved glycemic control [18]. Understanding the intricate molecular mechanisms underlying the impact of TZDs, such as pioglitazone, rosiglitazone, and lobeglitazone, on ketoacidosis provides valuable insights for clinicians seeking to optimize treatment strategies in diabetic patients prone to this metabolic complication.

## Conclusions

The narrative review supports the safety and efficacy of lobeglitazone, a novel thiazolidinedione derivative. Effectively controlling blood sugar, cholesterol, and liver function, lobeglitazone is also utilized as a secondary treatment for NAFLD. In other situations, such as ischemic stroke and corneal fibroblasts, lobeglitazone exhibits potent results. Similar to pioglitazone and rosiglitazone, among other thiazolidinediones, lobeglitazone also demonstrates significant anti-diabetic effects. Side effects of lobeglitazone, such as weight gain, peripheral edema, and BMD, fell into tolerable bounds and did not necessitate hospitalization. In light of sex variations and the existence of hepatic impairment, there is no need to modify the dosage. In India, a postmarketing surveillance phase 4 trial for lobeglitazone is now underway. Additional research is required to offer details on the efficacy and safety of lobeglitazone and the long-term safety of lobeglitazone. More studies are needed on lobeglitazone's effects on DKA.
